# Response of Macrophyte Traits to Herbivory and Neighboring Species: Integration of the Functional Trait Framework in the Context of Ecological Invasions

**DOI:** 10.3389/fpls.2018.01981

**Published:** 2019-01-10

**Authors:** Lise Thouvenot, Benoit Gauzens, Jacques Haury, Gabrielle Thiébaut

**Affiliations:** ^1^German Centre for Integrative Biodiversity Research (iDiv) Halle-Jena-Leipzig, Leipzig, Germany; ^2^Institute of Biology, Leipzig University, Leipzig, Germany; ^3^Institute of Biodiversity, Friedrich Schiller University Jena, Jena, Germany; ^4^National Institute of Agricultural Research, UMR ESE, Rennes, France; ^5^Agrocampus Ouest, Rennes, France; ^6^ECOBIO, UMR 6553 CNRS, Université de Rennes 1, Rennes, France

**Keywords:** associational susceptibility/resistance, biological invasion, community assemblage, ecological strategy, functional traits, herbivory

## Abstract

With the increase in the number of introduced species each year, biological invasions are considered as one of the most important environmental problems for native biodiversity. In invaded habitats, the establishment of exotic plant species depends on the abiotic and biotic environment. Herbivores and neighboring plants (native or exotic) comprise an important part of the latter. Herbivores cause trophic and non-trophic damage to focal plants, which respond to herbivory by varying their different traits quantitatively (e.g., growth rate and biomass changes) and qualitatively (e.g., variation in morphological and chemical defenses strategies affecting plant palatability). Neighboring plant species also affect functional traits and the fitness of focal plant species, thus herbivore effects on a focal plant could also depend indirectly on the palatability and defensive traits of the neighboring species inside the community. Here, in a first step toward the integration of associational susceptibility/resistance theories in the field of ecological invasion, we performed a microcosm experiment to consider the effects of an exotic crayfish on the growth rate, morphological traits and damage level of three macrophytes (two exotic, one native) growing in pairwise combinations. We found that (i) the response to herbivore presence and to neighboring species identity seemed to be species specific, and (ii) crayfish enhance the fragmentation rate of the two exotic macrophytes *Ludwigia grandiflora* and *Egeria densa* in the presence of the native macrophyte *Myriophyllum spicatum*, which could indirectly facilitate their invasion success. Indeed, fragmentation can increase dispersal abilities of the exotic macrophytes considered in this study as they are able to generate new plants from their fragments. However, our results showed that the interaction herbivore-neighbor species was hardly significant. Our paper presents some first results on associational resistance/susceptibility and lays the foundation for developing a general framework that combines plant community ecology and biological invasion ecology to explain invasive species success.

## Introduction

Biological invasions are one of the most important environmental threats for native biodiversity (Sala et al., 2000; Murphy and Romanuk, 2014), with the number of introduced species continuously increasing at a global scale (Seebens et al., 2016). In invaded habitats, exotic species tend to interact more often with each other, potentially reciprocally affecting their colonization success (Green et al., 2011). Indeed, in these new ranges, the establishment of exotic plant species depends on the abiotic and biotic environment (Shea and Chesson, 2002), and thus is modulated by surrounding herbivores and neighboring plants that can be native, exotic or both.

Herbivores cause trophic damage (Bakker et al., 2016; Wood et al., 2016), and/or non-trophic, non-consumptive indirect damage on plants like uprooting or propagule production *via* stem fragmentation/cutting (Gherardi and Acquistapace, 2007). Plants species respond to herbivory by varying different traits (Karban and Myers, 1989; Baldwin and Preston, 1999; Karban et al., 1999; Tiffin, 2000; Gong and Zhang, 2014), quantitatively (e.g., growth rate and biomass changes) and qualitatively (e.g., variation of morphological and chemical defense traits affecting plant palatability). In consequence, herbivores modify plant biomass allocation and thus plant competitive abilities against exotic and native neighboring plant species (Crawley, 1989; Duffy and Hay, 2000). However, the plant neighborhood also affects these traits (Callaway et al., 2003) and some functional plant traits vary according to the distance from the neighboring plants (Bittebiere and Mony, 2015). For example, in terrestrial ecosystems, the presence of neighboring plant species at a distance ranging from 5 to 25 cm from a focal plant species could explain 70% of the variation of leaf dry matter content (LDMC) (Bittebiere and Mony, 2015), a functional trait which is particularly involved in plant palatability. Thus, neighboring plant species can directly affect functional traits and fitness of focal plant species, but herbivore effects on a focal plant could also indirectly depend on the palatability and defensive traits of the neighboring species inside the community (Barbosa et al., 2009).

Indeed, the associations of different plant species and their effects on plant–herbivore interactions in terrestrial ecosystems has received lot of attention during the last decades (Barbosa et al., 2009; Ruttan and Lortie, 2014; Underwood et al., 2014), but less is known in freshwater ecosystems. The association between an unpalatable (or highly defended) plant and a palatable (or less defended) plant species can have two outcomes for each species: either a resistance or a susceptibility to herbivory. Several theories are derived from these two main outcomes (Atsatt and O’Dowd, 1976; Wahl and Hay, 1995; Ruttan and Lortie, 2014). Among them, the Associational susceptibility hypothesis (Wahl and Hay, 1995; White and Whitham, 2000; Emerson et al., 2012) stipulates an overconsumption of the palatable plant by herbivores in the presence of an unpalatable plant species, which in return will be eaten less by herbivores. Furthermore, the damage to unpalatable species by herbivores could be reduced in the presence of palatable species (“Attractant-decoy hypothesis”; Atsatt and O’Dowd, 1976; Ruttan and Lortie, 2014), or increased due to the presence of highly palatable species in the surrounding area, thus increasing patch attractiveness, as stipulated by the “Shared doom hypothesis” (Wahl and Hay, 1995). So, the outcome of the plant–plant–herbivore interaction relates simultaneously to the palatability and defense degree of the plant species involved, but also depends on the selectivity of the herbivore.

In the broader context of biological invasions, theories related to associational resistance/susceptibility could help to explain the establishment success of invasive species and potentially invasional meltdowns (Simberloff and Von Holle, 1999). The simultaneous introduction of an exotic herbivore and exotic plants could also explain the dispersal and colonization abilities of invasive plant species. For example, Parker et al. (2006) have shown that the percentage of total plant cover or biomass of exotic plants was 52% higher in communities grazed by exotic herbivores than in communities grazed by native herbivores. Exotic herbivores generally promote co-adapted exotic plants from the same native range (Parker et al., 2006) by negatively affecting native plants (*via* these consumptive and non-consumptive effects) (Wood et al., 2016) and by positively affecting the colonization abilities of exotic plants by stem fragmentation and propagule dispersal (Thouvenot et al., 2017), for example. However, the interactive effects of the introduction of an exotic herbivore and an exotic plant, and their outcomes on native plant communities are still understudied.

To take the first step toward the integration of associational susceptibility/resistance theories in the field of ecological invasions, we performed a microcosm experiment to consider the effects of an exotic crayfish on the growth rate, morphological traits and damage levels of three macrophytes (two exotic, one native) growing in pairwise combinations. The Red Swamp Crayfish, *Procambarus clarkii* (Girard, 1852), native to south-central United States (Louisiana) and north-eastern Mexico, has been successfully introduced worldwide for commercial purposes. *P. clarkii* occupies a key position in invaded ecosystems, with dramatic impacts on ecosystem structure and functions (Gutiérrez-Yurrita et al., 1998; Alcorlo et al., 2004). This species is an omnivore with plants as the first food source (88.45% of occurrence in the stomachs of the crayfish studied) followed by animals (59.16%) and detritus (31.19%) (Gutiérrez-Yurrita et al., 1998).

Overall, we tested (1) whether plant response depends on the neighboring plant species in the presence of an exotic crayfish (associational susceptibility vs. associational resistance) and (2) whether there is a facilitative effect of exotic crayfish on exotic plant dispersal.

## Materials and Methods

### Biological Material

*Procambarus clarkii* was collected in the Brière marshes (02° 18′ 53.3″ O, 47° 23′ 39.5″ N) in the Loire area, France. This species was chosen because of its abundance in marshes and ponds in north-western France and its high impact on the macrophyte community (Chambers et al., 1990; Gherardi and Acquistapace, 2007; Souty-Grosset et al., 2016). Only adult males with lengths ranging from 60 to 99 mm were selected to avoid sexual and maturity biases. Crayfish were starved in tap water for 1 week at ambient temperature.

Three macrophytes species differing in their morphology, origin and palatability to herbivores were chosen (Table 1): the native species with low palatability *Myriophyllum spicatum*, the exotic and highly palatable species *Egeria densa* and the exotic *Ludwigia grandiflora* with low palatably. The submerged shoots of each macrophyte species were collected in the field in Brittany either from ponds at Apigné (01° 44′ 25.2″ O, 48° 05′ 41.4″ N) or from the Gannedel marshlands (01° 56′ 09.6″ O, 47° 41′ 49.7″ N). These sites were selected as no crayfish species had been recorded, in order to avoid co-evolutionary adaptations. Shoots of plants species were stored for acclimatization in tap water for 1 week at ambient temperature.

**Table 1:** Characteristics (common name, family, biological type, status, habitat, and morphology) of the three aquatic plant species used in mixed cultures: *Egeria densa*, *Ludwigia grandiflora*, and *Myriophyllum spicatum*.

	*Egeria densa*	*Ludwigia grandiflora* subsp. *hexapetala*	*Myriophyllum spicatum*
Common name	Brazilian waterweed	Water primrose	Eurasian watermilfoil
Family	Hydrocharitaceae	Onagraceae	Haloragaceae
Biological type	Submerged freshwater plant	Amphibious freshwater plant	Submerged freshwater plant
Status/native area	Exotic in some European countries, Australia, New Zealand, Turkey/Native to South America^1^ .		Native to Europe, Asia, and Northern Africa/ Exotic in the United-States, Australia, South Africa, India^6^
Habitat	Still and flowing waters, lakes, ponds, pools and quiet streams.	Marshes, ponds, slow-running rivers, as well as wet meadows^3^.	Slow moving or still eutrophic water^7^.
Morphology	Dense monospecific stands Very bushy plant with dense whorls of robust leaves Four leaves per whorl and each leaf is at least 2 cm long. Palatable aquatic macrophyte^2^	Creeping submerged stems (glabrous to sparsely pubescent) and aerial shoots. Alternate, polymorphic^4^ leaves Reported as an unpalatable plant, whereas cases of grazing have been observed^5^	Stems grow to water surface and frequently form dense mats. Mature leaves: typically arranged in whorls of four leaves. Leaf has 12 or more leaflet pairs. Low palatable plant^8^: production of polyphenols^9^.


*^1^Cook and Urmi-König, 1985; Dutartre et al., 2007; Hussner, 2009; Thouvenot et al., 2013, ^*2*^Parker et al., 2006, ^*3*^Lambert et al., 2010, ^*4*^European and Mediterranean Plant Protection Organization, 2011, ^*5*^Pine and Anderson, 1991; Grillas et al., 1992; Legrand, 2002; Lambert et al., 2009, ^*6*^Weyl and Coetzee, 2014, ^*7*^Aiken et al., 1979, ^*8*^Li et al., 2004, ^*9*^Gross, 2000.*

### Experimental Design

To test our hypotheses, we set-up a greenhouse microcosm experiment in July 2011, with two crayfish treatments, with and without (control) crayfish, assigned to each of the different pairwise combinations of plants, in order to measure the effects of the exotic crayfish on the development of these macrophytes and the outcome of the plant–plant–crayfish interactions. For this purpose 24 containers were divided into experimental units (*L* ×*W* ×*H*: 33 cm × 40 cm × 35 cm) with an opaque plastic barrier that was impermeable to water. Each experimental unit was filled to 15 cm with water and contained 5 cm of sand as well as two patches of two different species: either *L. grandiflora*/*E. densa* or *L. grandiflora*/*M. spicatum* or *M. spicatum*/*E. densa*. Each plant patch was planted in each half of each experimental unit and corresponded to three shoots of one plant species (with a total fresh biomass per species ranging from 2.3 to 3.3 g). Each shoot corresponded to a stem fragment (length ranging from 6 to 12.5 cm) with an apical apex and without roots or lateral buds. Only shoots with green leaves without any sign of grazing or necrosis were used for the experiment. In addition to the control treatment (without crayfish), a crayfish treatment (with one crayfish) was set-up in order to evaluate both the trophic and non-trophic damage to plants. The density of crayfish per aquaria (corresponding to ≈ 7.6 ind.m^-2^) was chosen to be in the range of the densities recorded in invaded natural areas. For example, around 3.8 ind.m^-2^ were trapped in a Spanish wetland (Angeler et al., 2001) while 14 ind.m^-2^ were recorded in a Mediterranean wetland (Scalici and Gherardi, 2007). Each treatment with each plant pair was replicated eight times. The six combinations were randomly assigned to the different containers. The chemical composition of the tap water was analyzed at the beginning of the experiment using spectrophotometric techniques (WTW kit and Photolab S12) (mean value: pH = 8.47; conductivity: 618 μS/cm; [O_2_] = 10.47 ± 0.24 mg.L^-1^; [NO_2_^-^] = 0.13 ± 0.02 mg.L^-1^; [NO_3_^-^] = 14.82 ± 0.51 mg.L^-1^; [NH_4_^+^] = 0.11 ± 0.02 mg.L^-1^; [PO_4_^3-^] = 0.15 ± 0.02 mg.L^-1^). During the experiment, the water was not aerated, no nutrients were added, and the water levels in the containers were maintained regularly with tap water. The light intensity was natural and the temperature was measured every half minute with three sensors (HOBO TidbiT Water Temperature Data Logger). The water temperature in the glasshouse (means ± SD: 20.96 ± 0.05°C) was similar to those found in the summer in the channels of the Brière marshes. The experiment was stopped after 3 days, when the biomass of one macrophyte species was reduced by half, allowing us to measure traits and make comparisons with the literature (Cronin et al., 2002; Anastacio et al., 2005; Carreira et al., 2014). All plant fragments were removed, and plant traits were measured at the end of the experiment.

### Plant Traits Analyses

Six morphological traits were measured on plants. Plant growth, an indicator of tolerance to herbivory (Coley et al., 1985; Agrawal, 2011), was evaluated by the Relative Growth Rate (RGR) adapted from Hunt (1990): *RGR* = *(ln B2 – ln B1)/(T1-T2)*, where *B1* and *B2* refer to the total fresh biomass of the three fragments at time 1 and time 2 (biomasses only considered the growth of the original fragments and excluding cut shoots that appeared during the experiment). We quantified the damage to plants induced by crayfish by measuring the mean percentage of damaged leaves per shoot (leaves with scars or holes) and the free leaf biomass. Free leaf biomass consisted of only the leaves cut by the crayfish and found in the water column or floating at the water surface in the aquarium. When a shoot fragment had disappeared from the aquarium, we included a number of 100% of damaged leaves for this shoot in the calculation. As plants fragments of *E. densa*, *M. spicatum*, and *L. grandiflora* are able to regenerate new plants (Hussner, 2009; Riis et al., 2009; Heidbüchel et al., 2016), we assumed that crayfish could have a positive impact on the invasive success of exotic plants by enhancing their dispersal due to the increase in shoots cut by crayfish. We quantified this impact on plant dispersal by using the mean number of additional fragments, which was calculated as the number of shoots at the end of the experiment, less this number at the beginning. To evaluate the palatability of the plant species, we measured the dry matter content (DMC) and LDMC. A low water content (i.e., high DMC) and high concentrations of proteins and nitrogen in plants are associated with a high nutritive value (Cronin et al., 2002). The LDMC is used as a proxy to predict variations in macrophyte palatability (Elger and Willby, 2003): it is related to the average density of leaf tissues (Cornelissen et al., 2003) and leaf constituents such as lignin, fiber, and silica contents which contribute to leaf toughness (Elger and Willby, 2003) and to the morphological defenses of plants. Leaves and shoots were collected, weighed (fresh mass), dried (for 1 week at 70°C) and then reweighed (dry mass). Prior to drying, fresh leaves of the invasive species *L. grandiflora* and *E. densa* were scanned and leaf area was measured using Scion Image software in order to calculate the specific leaf area (SLA). Leaf area of *M. spicatum* was not calculated as its leaves are small, thin and highly dissected. DMC (g.g^-1^) was assessed for each pool of three shoots per species, using the dry mass of the pool divided by the fresh mass of the pool of individuals. The LDMC (mg.g^-1^), calculated as dry mass of the leaf divided by its fresh mass, was measured on three leaves attached to each planted shoot (upper part of the plant shoot). SLA (mm^2^.mg^-1^) which is correlated to relative growth rate and investment in structural tissue was calculated as the ratio of fresh leaf area to leaf dry mass (mm^2^ mg^-1^). Data of SLA and LDMC were averaged per stem.

### Statistical Analysis

We tested the associational resistance and susceptibility hypotheses by performing a two-factor Bayesian ANOVA per species. ANOVA was defined as a linear model with the identity of the neighboring species (two categories in each analysis), the crayfish (presence or absence) and their interaction as factors. To account for heteroscedasticity, we used an additional parameter to estimate within factor variability. When several measurements were performed on the same individual (here, traits related to leaves: SLA and LDMC), we built a mixed effect model, with a specific error term to consider individuals as a random factor. Logarithmic transformations were used for SLA and LDMC.

As there was no variability in the control treatment for the free leaf biomass, the proportion of damaged leaves and the number of cut shoots due to the absence of crayfish, the damage induced by the presence of the crayfish was tested for each species by comparing posterior distributions of the mean to zero. Then, the influence of neighboring species was analyzed only for treatments with crayfish. We tested the diet preference of crayfish by doing pairwise comparisons of posterior distributions of the herbivory effect on the RGR for each plant species.

Posterior effect size distributions for simple effects were computed as contrast (a difference in posterior mean distributions for the two levels of one factor). Effect sizes for pairwise comparisons were computed as the difference in posterior distributions of the considered treatments. Significance of effects was defined as the probability of effect sizes (posterior distributions for the interaction term) being lower or greater than zero. We used a threshold of significance equal to 0.05 (thereafter we speak of tendencies when we used a threshold of 0.1). We chose non-informative priors for each of the model parameters. Model parameters were estimated by Markov chain Monte Carlo sampling (MCMC) with the rags 4.3.0 library in R 3.4.4. We ran four independent chains of 50,000 iterations with a burn-in period of 20,000. Quality of model fit was assessed using Gelman–Rubin diagnostics. Untransformed means and standard errors were used in the figures to facilitate interpretation. Several points appeared to have a significant impact on the conclusions of the statistical analyses. Outliers detected using an approach based on the Cook distance were removed from analyses (see Supplementary Materials for more details on the procedure). When data points were removed, we present the output of our models with and without these data points in the Results section. All analyses were performed with R software (R Core Team, 2017). Codes used for data analyses are accessible on GitHub at https://github.com/gauzens/traits_and_invasions.git.

## Results

### Crayfish Selectivity

By comparing differences in RGR induced by herbivory, we found that *E. densa* was consumed more than *L. grandiflora* and *M. spicatum*. Indeed, posterior distribution of the herbivory effect on *E. densa* was significantly smaller than the one found for *L. grandiflora* (*p* = 0.002) and *M. spicatum* (*p* = 0.0195). We did not find a significant difference in selectivity between *L. grandiflora* and *M. spicatum* (*p* = 0.271).

### Effect of Neighborhood and Herbivory on the More Palatable Exotic Plant *E. densa*

**FIGURE 1:**
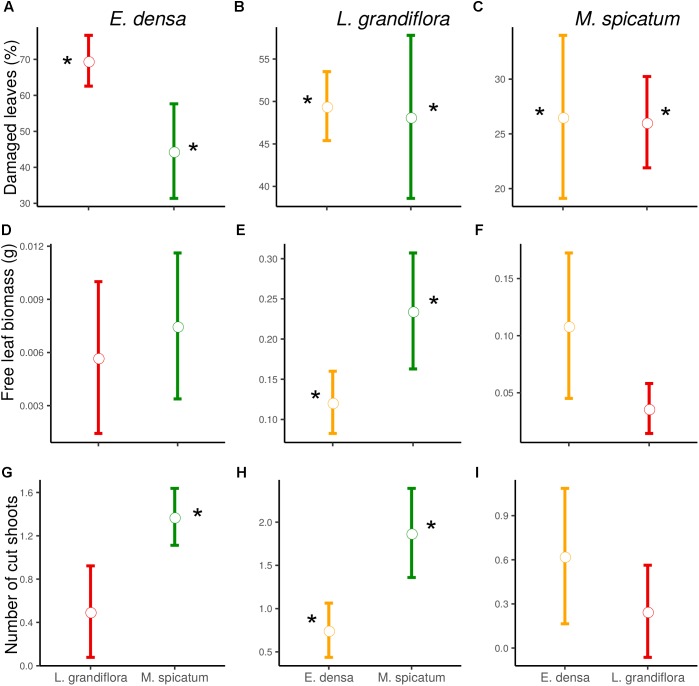
Effect of neighboring species and herbivory on the damaged leaves **(A–C)**, free leaf biomass **(D–F)**, and number of cut shoots **(G–I)** of *E. densa*, *L. grandiflora* and *M. spicatum* (Means ± SE). Colors code the identity of the neighboring species: orange for *E. densa*, red for *L. grandiflora* and green for *M. spicatum*. Stars show the significant herbivory effects (significance threshold of 0.05).

The functional traits of the exotic *E. densa* mainly depended on the presence of crayfish, and were marginally affected by the neighboring species (Tables 2, 3). Its proportion of damaged leaves increased significantly because of herbivory in presence of *M. spicatum* (*p* = 0.037) and *L. grandiflora* (*p* < 0.001, Table 3 and Figure 1). *E. densa* tended to lose more leaf biomass (*p* = 0.077) and was more fragmented (*p* = 0.001) due to severing, especially in the presence of *M. spicatum* as a neighbor species, while these effects were not detected in the presence of *L. grandiflora* (Table 3 and Figure 1). However, we only detected a marginal difference in fragmentation between the two neighboring species (*p* = 0.081).

**Table 2:** Summary of two-factor Bayesian ANOVAs performed for each species and each measured trait: relative growth rate (RGR), dry matter content (DMC), leaf dry matter content (LDMC), and specific leaf area (SLA).

	**RGR (d^-1^)**		**DMC (g.g^-1^)**		**LDMC (mg.g^-1^)**		**SLA (mm^2^.mg^-1^)**	
*E. densa*								
Neighboring species (N)	0.032	(0.288)	*0.792*	*(0.092)*	0.055	(0.234)	0.067	(0.267)
Crayfish treatment (C)	**0.266**	**(<0.001)**	0.156	(0.399)	0.095	(0.107)	0.116	(0.128)
Interaction (N) × (C)	0.069	(0.268)	0.917	(0.400)	0.230	(*0.068)*	**0.356**	**(0.040)**
*L. grandiflora*								
Neighboring species (N)	0.009	(0.320)	0.135	(0.377)	*0.041*	(*0.074*)	**0.084**	**(0.001)**
Crayfish treatment (C)	**0.049**	**(0.022)**	0.107	(0.398)	0.016	(0.280)	0.012	(0.319)
Interaction (N) × (C)	0.041	(0.173)	0.022	(0.496)	*0.088*	(*0.063*)	0.014	(0.393)
*M. spicatum*								
Neighboring species (N)	0.005	(0.465)	1.261	(0.146)	**0.099**	**(0.048)**	/	
Crayfish treatment (C)	*0.088*	*(0.067)*	0.730	(0.271)	0.056	(0.196)	/	
Interaction (N) × (C)	*0.156*	*(0.088)*	0.857	(0.352)	0.072	(0.299)	**/**	


*Values presented are effect sizes of the different factors estimated from the posterior distributions and associated *p*-values within brackets. Significant results are in bold type, and tendencies are in italics.*

**Table 3:** Summary of two-factor Bayesian ANOVAs performed for each species and each type of damage induced by crayfish: percentage of damaged leaves, free leaf biomass and number of cut shoots.

Species	*E. densa*		*L. grandiflora*		*M. spicatum*	
Neighboring species	*M. spicatum*	*L. grandiflora*	*E. densa*	*M. spicatum*	*E. densa*	*L. grandiflora*
**Damaged leaves (%)**						
Neighboring species (N)	0.254 (0.123)		0.014 (0.458)		0.004 (0.490)	
Crayfish treatment (C)	**0.442 (0.037)**	**0.6959 (<0.001)**	**0.496 (<0.001)**	**0.481 (0.002)**	**0.265 (0.009)**	**0.260 (0.002)**
**Free leaf biomass (g)**						
Neighboring species (N)	0.002 (0.409)		0.113 (0.125)		0.071 (0.195)	
Crayfish treatment (C)	*0.007 (0.077)*	0.005 (0.134)	**0.122 (0.011)**	**0.235 (0.010)**	*0.108 (0.092)*	*0.037(0.082)*
**Number of cut shoots**						
Neighboring species (N)	*0.877* (*0.081*)		*1.139* (*0.071*)		0.359 (0.297)	
Crayfish treatment (C)	**1.382 (0.001)**	0.504 (0.158)	**0.746 (0.038)**	**1.88 (0.008)**	0.614 (0.136)	0.255 (0.237)


*Values presented are effect sizes of the different factors estimated from the posterior distributions and associated *p*-values within brackets. Significant results are in bold type, and tendencies are in italics.*

**FIGURE 2:**
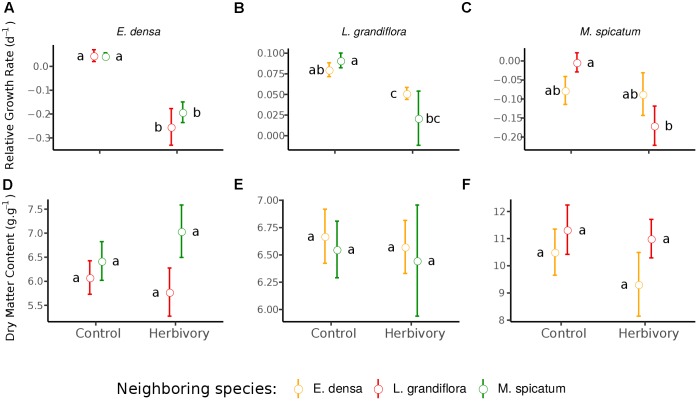
Effect of neighboring species and herbivory on the relative growth rate **(A–C)** and dry matter content **(D–F)** of *E. densa*, *L. grandiflora* and *M. spicatum* (Means ± SE). Colors code the identity of the neighboring species: orange for *E. densa*, red for *L. grandiflora* and green for *M. spicatum*. Letters indicate the significance of pairwise comparisons (significance threshold of 0.05).

**FIGURE 3:**
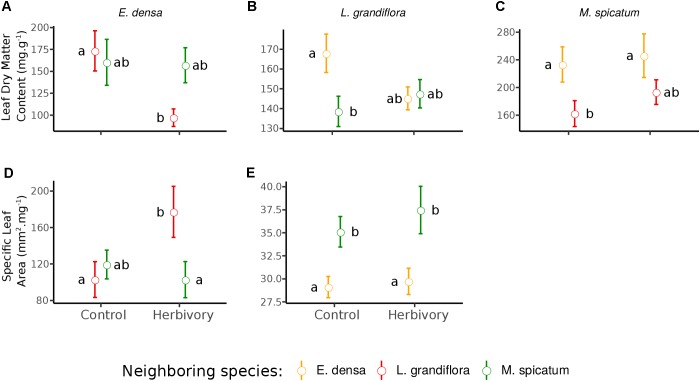
Effect of neighboring species and herbivory on the leaf dry matter content **(A–C)** and specific leaf area **(D,E)** of *E. densa*, *L. grandiflora*, and *M. spicatum* (Means ± SE). Colors code the identity of the neighboring species: orange for *E. densa*, red for *L. grandiflora* and green for *M. spicatum*. Letters indicate the significance of pairwise comparisons (significance threshold of 0.05).

The RGR of *E. densa* was significantly reduced by herbivory from crayfish (*p* < 0.001), but it was not affected by the identity of the neighboring species (*p* = 0.288). No significant interaction between these two factors was found (*p* = 0.268, Figure 2 and Tables 2, 4). For the DMC of *E. densa*, we found that one data point was quite influential (five times larger than the mean value of its group) and led to the absence of any effects. Once removed, the DMC of *E. densa* tended to be higher in the presence of *M spicatum* than in the presence of *L. grandiflora* (*p* = 0.092, Table 2 and Figure 2), and mainly in the crayfish treatment as shown by the pairwise comparison between treatment crayfish-*L. grandiflora* and crayfish-*M. spicatum* (Table 4), while the DMC of *E. densa* was similar in the control treatment, whatever the neighboring species. The LDMC was marginally affected by the interaction crayfish-neighboring species with values tending to be higher (*p* = 0.068, Table 2 and Figure 3) in the presence of *M. spicatum*. This is most likely to be explained by the significant decrease of LDMC due to crayfish in presence of *L. grandiflora* (*p* = 0.024, Table 4 and Figure 3), while we did not detect any effect of crayfish in the presence of *M. spicatum* on LDMC. We observed a significant interaction between the crayfish treatment and the identity of the neighboring species for the SLA (*p* = 0.040, Table 2 and Figure 3), explained by a significantly higher SLA in the presence of *L. grandiflora* in comparison with values observed in the presence of *M. spicatum* for crayfish treatments (*p* = 0.050, Table 4), while the SLA of *E. densa* was similar in control treatments whatever the identity of the neighboring species (*p* = 0.209, Table 4).

**Table 4:** Summary of the pairwise comparisons performed for each species and each measured trait: relative growth rate (RGR), dry matter content (DMC), leaf dry matter content (LDMC), and specific leaf area (SLA).

	**RGR (d^-1^)**		**DMC (g.g^-1^)**		**LDMC (mg.g^-1^)**		**SLA (mm^2^.mg^-1^)**	
*E. densa*								
Crayfish, *L. grandiflora* – Control, *L. grandiflora*	**–0.300**	**(0.006)**	–0.303	(0.344)	**–0.210**	**(0.024)**	**0.241**	**(0.029)**
Crayfish, *L. grandiflora –* Crayfish, *M. spicatum*	–0.066	(0.263)	*–1.251*	*(0.094)*	*–0.171*	*(0.068)*	**0.241**	**(0.050)**
Crayfish, *L. grandiflora –* Control, *M. spicatum*	**–0.300**	**(0.006)**	–0.637	(0.206)	*–0.150*	*(0.086)*	0.179	(0.114)
Control, *L. grandiflora –* Crayfish, *M. spicatum*	**0.235**	**(0.003)**	–0.948	(0.129)	0.039	(0.361)	0.0003	(0.360)
Control, *L. grandiflora –* Control, *M. spicatum*	0.002	(0.474)	–0.334	(0.298)	0.060	(0.282)	–0.062	(0.209)
Control, *M. spicatum –* Crayfish, *M. spicatum*	**–0.230**	**(0.001)**	0.614	(0.226)	0.020	(0.418)	–0.062	(0.312)
*L. grandiflora*								
Crayfish, *E. densa –* Control, *E. densa*	**–0.029**	**(0.021)**	–0.096	(0.411)	*–0.061*	*(0.071)*	0.004	(0.442)
Crayfish, *E. densa –* Crayfish, *M. spicatum*	0.030	(0.223)	0.146	(0.413)	–0.003	(0.474)	**–0.092**	**(0.007)**
Crayfish, *E. densa –* Control, *M. spicatum*	**–0.040**	**(0.005)**	0.028	(0.473)	0.025	(0.249)	**–0.073**	**(0.015)**
Control, *E. densa –* Crayfish, *M. spicatum*	*0.059*	*(0.082)*	0.242	(0.366)	*0.058*	*(0.090)*	**–0.096**	**(0.006)**
Control, *E. densa –* Control, *M. spicatum*	–0.011	(0.220)	0.123	(0.402)	**0.086**	**(0.022)**	**–0.077**	**(0.008)**
Control, *M. spicatum –* Crayfish, *M. spicatum*	*–0.070*	*(0.053)*	–0.118	(0.433)	0.028	(0.247)	0.019	(0.311)
*M. spicatum*								
Crayfish, *E. densa –* Control, *E. densa*	–0.008	(0.466)	–1.158	(0.256)	–0.008	(0.467)	/	
Crayfish, *E. densa –* Crayfish, *L. grandiflora*	0.082	(0.196)	–1.690	(0.158)	0.061	(0.255)	/	
Crayfish, *E. densa –* Control, *L. grandiflora*	–0.082	(0.147)	–1.991	(0.142)	*0.156*	*(0.053)*	/	
Control, *E. densa –* Crayfish, *L. grandiflora*	0.090	(0.130)	–0.532	(0.342)	0.069	(0.215)	/	
Control, *E. densa –* Control, *L. grandiflora*	*–0.074*	*(0.089)*	–0.833	(0.294)	**0.163**	**(0.040)**	/	
Control, *L. grandiflora –* Crayfish, *L. grandiflora*	**–0.165**	(**0.017**)	–0.301	(0.411)	0.094	(0.164)	/	


*Values presented are effect sizes of the different factors estimated from the posterior distributions and associated *p*-values within brackets. Significant results are in bold type, and tendencies are in italics.*

### Effect of Neighborhood and Herbivory on the Less Palatable Exotic Plant *L. grandiflora*

In contrast to the palatable exotic species and despite the different types of damage induced by crayfish, the RGR of *L. grandiflora* remained positive under crayfish pressure. Whatever the neighboring species, the free leaf biomass of *L. grandiflora* found in the water column significantly increased in the presence of crayfish (in association with *M. spicatum*: *p* = 0.010, *E. densa:* *p* = 0.011) and its leaves were highly damaged (in the presence of *M. spicatum*: *p* = 0.002, *E. densa*: *p* < 0.001, Table 3 and Figure 1). Similarly, the presence of crayfish induced an increase in stem fragmentation of *L. grandiflora* (in the presence of *M. spicatum*: *p* = 0.008, *E. densa*: *p* = 0.038, Table 3 and Figure 1), which tended to be higher in the presence of the native species *M. spicatum* (*p* = 0.071) in comparison with the association with *E. densa*.

Furthermore, the RGR of *L. grandiflora* depended on crayfish presence but not on the neighboring species. Crayfish presence reduced the RGR of *L. grandiflora* (*p* = 0.022, Figure 2 and Table 2), particularly when this species grew up with the exotic species *E. densa* (*p* = 0.021, Table 4). This effect became marginal in the presence of *M. spicatum* (*p* = 0.053, Table 4) due to a stronger size effect, corresponding to a large variability of RGR values observed for treatments with crayfish and *M. spicatum*. SLA was significantly affected by the identity of the neighboring species (*p* = 0.001, Table 2 and Figure 3), values always being significantly higher in the presence of *M. spicatum*. We did not observe any effect of crayfish presence (*p* = 0.319) nor interaction (*p* = 0.393, Table 2) on SLA of *L. grandiflora*. When we performed analyses including the three data points from the experimental unit removed, we only found a marginal interaction effect (*p* = 0.080), as the mean SLA value for the control treatment in the presence of *E. densa* increased drastically from 29.11 mm^2^.mg^-1^ (the smallest observed mean within all treatments) to 50.12 mm^2^.mg^-1^ (the largest one), thus obscuring the crayfish effect. The DMC of *L. grandiflora* was not impacted by the different treatments nor their interaction (Table 2), but we found a marginal interactive effect on LDMC (*p* = 0.063, Table 2 and Figure 3), because of a significant impact of neighboring species in the control (larger values when associated to *E. densa*, *p* = 0.022, Table 4) becoming not significant in crayfish treatments (*p* = 0.474, Table 4). When we performed the analyses including the data points of the experimental unit initially removed, all the results became not significant.

### Effect of the Exotic Neighboring Plant and the Exotic Herbivore on the Less Palatable Native Plant *M. spicatum*

The native *M. spicatum* was barely affected by the crayfish and neighboring species, although these factors or their interaction tended to affect some of its traits. We did not observe any effect of the neighboring species on the damage to *M. spicatum*; the only significant differences were related to the presence of crayfish (Table 3 and Figure 1). The number of cut shoots was not affected by crayfish presence or by the neighboring species. The percentage of damaged leaves increased in the presence of crayfish (*p* = 0.002 when associated with *L. grandiflora*, *p* = 0.009 when associated to *E. densa*; Table 3), but we did not find a significant (only a marginal) effect of crayfish on the free leaf biomass lost by *M. spicatum* (*p* = 0.082 in presence of *L. grandiflora* and *p* = 0.092 in presence of *E. densa*, Table 3).

The RGR of *M. spicatum* tended to decrease in the presence of crayfish (*p* = 0.067, Table 2 and Figure 2), this effect being significant in the presence of *L. grandiflora* (*p* = 0.017, Table 4), but disappearing in the presence of *E. densa* (*p* = 0.466, Table 4) explaining a marginal interaction (*p* = 0.088, Table 2). When the outlier was included in our analysis, the only qualitative change was for the interaction term (*p* = 0.427). The DMC of *M. spicatum* was not affected by the neighboring species, crayfish presence or their interaction (Table 2 and Figure 2). We observed a significant effect of neighboring species on the LDMC (*p* = 0.048, Table 2): the values of the LDMC of *M. spicatum* were higher in the presence of *E. densa* than in the presence of *L. grandiflora* in control treatments but not in the presence of crayfish (*p* = 0.040, Table 4).

## Discussion and Perspectives

### Plant Response to the Neighboring Plant Species and the Herbivore

Crayfish caused significant damage to the plant species, as shown by the systematic decrease of RGR and the increase of damaged leaves, and as reported by many authors (Flint and Goldman, 1975; Feminella and Resh, 1989; Sánchez and Angeler, 2006). However, the absence of significant crayfish effects on floating free leaf biomass and number of cut shoots (floating plus rooted) observed for *M. spicatum* and to a lesser extend for *E. densa* could be explained by the ingestion of these floating plant organs by the crayfish, which were consequently not collected in the water column at the end of the experiment. This hypothesis could be verified in further short-term experiments by checking crayfish feeding behavior using video tracking.

**FIGURE 4:**
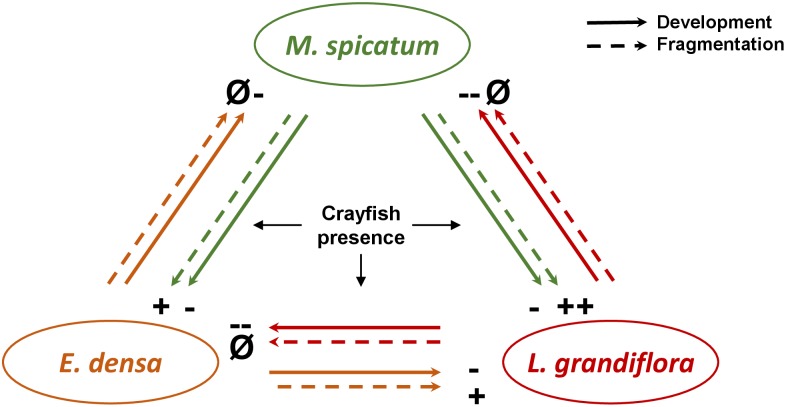
Schematic diagram of the effects of interactions between the different plant species (the native species *M. spicatum*, and the exotic species *E. densa* and *L. grandiflora*) under herbivore pressure on the development (RGR, LDMC) and fragmentation (number of cut shoots) of the plant species involved in the experiment. The effects are positive (+), negative (–) and neutral (i.e., without effect, Ø).

In this experiment, we studied plant traits in the context of the interactive effect of neighboring species and herbivore pressure that had rarely been considered before. Overall, the effects of the crayfish treatment and of the neighboring species seemed to be species specific. We did not find general patterns of plant responses (SLA, LDMC, etc.) or of herbivory damage (number of cut shoots, percentage of damaged leaves, free leaf biomass) to neighboring species and herbivory, suggesting that the three plants considered follow different strategies regarding crayfish presence and the identity of the neighboring species. Indeed, the response of plant traits (Figure 4) and especially plant palatability (regarding SLA and LDMC) were only modestly affected by the interaction between neighboring species and the herbivore, but rather by the identity of neighboring species or the herbivore effect alone. SLA is a function of LDMC and leaf thickness (Cornelissen et al., 2003; Pérez-Harguindeguy et al., 2013) and consequently it would explain the trends observed regarding the neighboring effect on the LDMC of *L. grandiflora*, and the interaction effect of neighboring species and crayfish on the LDMC of *E. densa* (Table 4).

The SLA values of *E. densa* depended on the interaction neighboring species - crayfish treatment. In the presence of crayfish, high SLA values were observed when the neighboring species was *L. grandiflora*, while no variation was observed in the presence of *M. spicatum*. This variation observed under herbivore pressure for *E. densa* could have two explanations. Firstly, it might be a direct effect of the consumption of younger leaves (with high SLA and low LDMC) by herbivores. Secondly, this difference can be explained by a morpho-physiological response of *E. densa*, suggesting that macrophytes species could show a rapid adaptation in response to herbivory. As we observed changes of the SLA of *E. densa* only in presence of *L. grandiflora* (despite a significant herbivory with the two neighboring species), the first explanation is rather unlikely. Moreover, several examples showing that macrophytes can exhibit morphological responses to stressors are documented, arguing for our second hypothesis. For example, *Rumex palustris* can increase its leaf laminae in response to submergence within 3 days (Voesenek and Blom, 1989). Morphological responses were also observed in 3 days for alga *Padina jamaicensis* in response to a reduction of grazing intensity (Lewis et al., 1987). Fast changes in leaf orientation and coloration can be observed after 24 h of herbivory on *M. spicatum* (Fornoff and Gross, 2014) and this species increased its DMC in its apices after 5 days under herbivore pressure (Fornoff and Gross, 2014). The DMC of *Elodea canadensis* and *Elodea nuttallii* were also higher in the presence of the herbivore after 6 days of experiment (Thiébaut et al., 2017). In the specific case of *E. densa*, it has been shown that this species has high growth abilities, with a RGR of up to 37–40 mg g^-1^ day^-1^ (Tanner et al., 1993), or from 0.03 to 0.05 g g day^-1^ of dry mass (Pistori et al., 2004). This strong growth rate suggests that *E. densa* could have a high ability to respond to damage through fast leaf/stem elongation. With the increase in SLA of *E. densa* (and thus indirectly to photosynthetic activity), we observed more allocation of energy to growth and not to structural tissues (which would imply an increase of LDMC) in the presence of *L. grandiflora*, potentially leading to more brittle and appetizing plant individuals of *E. densa*. This result suggests that *E. densa* allocated its resource to growth in order to compensate herbivore damage (Strauss and Agrawal, 1999). Furthermore, Lemoine et al. (2009) have shown that *M. spicatum* and *Elodea canadensis* reduced their DMC in response to herbivores. The reduction of DMC is associated with a reduction of the cell wall resistance (Gatehouse, 2002; Lemoine et al., 2009) which is positively correlated to the fragmentation ability of plants (Gatehouse, 2002) and could be considered as an escape strategy against herbivory. *E. densa*, which is a species phylogenetically close to *E. canadensis*, had a higher SLA and seemed to have a lower DMC and LDMC in the presence of *L. grandiflora* and crayfish (Figure 2 and Tables 2, 4) than in the presence of *M. spicatum*, while no variations were observed in the control treatments. Thus, this strategy suggests that under herbivore pressure, *E. densa* favors its fragmentation and dispersal abilities in the presence of the unpalatable plant species *L. grandiflora*.

In the case of *M. spicatum* and *L. grandiflora*, SLA and LDMC values were different in the control treatments, with higher LDMC values and lower SLA values (SLA only for *L. grandiflora*) being found when they were grown in association with the palatable species *E. densa*. These differences in LDMC completely disappeared in the presence of crayfish for *L. grandiflora*, and tended to decrease for *M. spicatum*. In the meantime, the responses of their RGR values were rather similar: the RGR of both species tended to decrease in the presence of an unpalatable neighbor, while the impact of crayfish was much lower in the presence of the palatable species *E. densa* (nearly no herbivory effect for *M. spicatum* with *E. densa*). Plants with high LDMC and low SLA generally possess strong quantitative leaf defenses and deter herbivores (Cornelissen et al., 2003; Kirk et al., 2012), while a high SLA and low LDMC are correlated with a high relative growth rate, but low resistance to herbivory (Cornelissen et al., 2003). Thus these two species seem to have similar responses to the presence of a neighbor species (high investment in defense in the presence of a palatable species), but this strategy is blurred in the presence of herbivores. The disappearance of the impact of a neighbor effect in the presence of crayfish would suggest that in our experiment the stress induced by herbivory was much stronger than the interaction with neighbors. As both *M. spicatum* and *L. grandiflora* are able to produce defensive compounds (Gross, 2000; Dandelot et al., 2008; Bauer et al., 2009; Lemoine et al., 2009), it is possible that the ecological strategy of these two species is toward other defense mechanisms to herbivory that were not considered here (i.e., secondary compounds involved in chemical defenses).

Considered together, our results highlight that the variation of SLA and LDMC could reflect anti-herbivore (*E. densa*) and/or anti-neighbor strategy (*L. grandiflora*, *M. spicatum*) during the time of our experiment (3 days). However, we still do not know how fast the plant traits response to herbivory or neighboring species could be. This calls for further experiments with time-series sampling to reinforce or question our conclusions. In addition, our results also showed that plant traits were affected by herbivory but also by an interaction with neighboring species, suggesting that these two ecological processes interact, and call for further investigations on underlying mechanisms and ecological consequences.

### Consequences of Plant Strategy on the Outcomes of the Plant–Plant–Herbivore Relationship

Plant traits influence plant performance and explain their growth and survival across different ecosystems. The set of the different trait values and their combinations represents alternative ecological strategies of the plant species in response to variations of environment. Considering the overall responses of all the traits of each species to its neighbors and under crayfish pressure would make it possible to assess the outcomes of these plant–plant–herbivore interactions. Below, we summarize the different effects observed on the traits of each species in order to deduce the potential outcomes (Figure 4).

#### E. densa

The reduction of *E. densa* growth due to herbivory was always stronger than that observed for the two other species. This is coherent with the high palatability of the species. The effect size of the herbivory effect was rather similar, whatever the associated neighboring plant was. However, in presence of *L. grandiflora*, the SLA of *E. densa* highly increased, potentially due to the reallocation of biomass to the apical growth, or due to the lower consumption of young parts of the plant. Thus, our results suggest that the youngest parts of *E. densa* would be less consumed in presence of *L. grandiflora* than in presence of *M. spicatum* and/or that *L. grandiflora* stimulated the growth of *E. densa* to compensate the loss due to crayfish. It would be interesting to test whether over a longer time period this compensation would attenuate the crayfish effect on the growth of *E. densa*.

#### L. grandiflora

Despite having a high number of cut shoots and a low SLA, *L. grandiflora* exhibited the lowest reduction of RGR under herbivore pressure. Thus, this species might be able to continue to acquire resources in order to tolerate crayfish consumption and to compensate the damage by its high growth rate. The effect of the herbivore was stronger in presence of *M. spicatum* despite the fact that the traits related to palatability remained similar whatever the identity of the neighboring species: this suggests more a dilution effect of crayfish impacts in the presence of *E. densa* than a higher susceptibility to crayfish damage in the presence of *M. spicatum*.

#### M. spicatum

Similarly, the growth of the native *M. spicatum* decreased under crayfish treatment whatever the identity of the neighboring species, with a higher effect in the presence of *L. grandiflora* than *E. densa*, also suggesting a dilution effect of herbivory on *M. spicatum* in the presence of the more palatable *E. densa*. *E. densa* could be an attractant-decoy plant, and consequently would decrease the crayfish damage on the neighboring species *M. spicatum*.

### Is There a Facilitative Effect of an Exotic Herbivore on Exotic Plant Development and Propagation in the Presence of a Native Plant?

Exotic crayfish promoted the propagation and the dispersal capacities of both exotic plants in the presence of the native plant during our experiment. Indeed, we observed that crayfish broke up each species, increasing the number of cut shoots.

The presence of the native *M. spicatum* in crayfish presence had an indirect positive effect on the invasive species *L. grandiflora* and *E. densa* by increasing their fragmentation rate. We can also suppose that the fragmentation of *E. densa* was increased in presence of *L. grandiflora*, explaining the high decrease of its RGR, but that the cut shoots were consumed by crayfish due to their higher appetence/palatability. As previously developed, fragmentation could be considered as an escape strategy (Lemoine et al., 2009) and could lead to plant propagation. Thus, this direct negative impact of crayfish in the presence of the native species could influence the rate at which an invasion occurs, with a positive effect on the dispersal and propagation of the exotic plant species in ecosystems.

Although the survival and anchorage rate of these cut floating shoots and their abilities to generate new plants are crucial points that remain to be investigated, the literature shows that most aquatic plants can regenerate new plants from their fragments (Pieczynska, 2003). Stem fragments of *L. grandiflora*, with nodes, and with or without leaves showed a high potential for regeneration (Hussner, 2009) and had a higher anchorage rate in the presence of the invasive species *E. canadensis* (Thiébaut and Martinez, 2015). Similarly, stem fragments of *E. densa* (1 cm in length) (Cook and Urmi-König, 1984; Getsinger and Dillon, 1984; Riis et al., 2009) and stem fragments (2 cm in length) of *M. spicatum* have a high regeneration capacity and can develop into new shoots, even without the presence of an apical bud (Riis et al., 2009; Kuntz et al., 2014; Heidbüchel et al., 2016).

### Associational Susceptibility/Resistance Theories in the Biological Invasions Context

A lot of attention has been paid to the defense strategies of plants against herbivory, however, little is known on how it will affect plant coexistence in the face of a combination of several biotic factors (competition with invasive species and herbivory in our paper). Our study showed that the effect of exotic crayfish and neighboring exotic plants influence each other. Therefore, these two factors could be important for the establishment of exotic species in new areas and for the structure and composition of new communities. As traits of neighboring species can affect the response of plants to herbivory, we could expect that exotic palatable species could modify the establishment and growth of other less palatable exotic species, and favor the disappearance of native palatable plants in an invaded area. Unfortunately, associational resistance and associational susceptibility were, to our knowledge, not tested in the context of biological invasions. This experiment is the first step to investigate this topic. By using one native species and two exotic species, we were not able to determine a general framework on the protective effects of exotic plants on native plants, as well as the protective effects of exotic plants on other exotic plants (or native on native plants), leading to a potential invasional meltdown (Simberloff and Von Holle, 1999) between exotic species. However, the different effects highlighted between exotic and native species in our study provide the first examples of such effects and call for further investigation, using different plants (target and neighboring species from native and exotic ranges) and herbivore species (native and exotic) to consider the generality of our findings. The foraging behavior and origin of the herbivore species also deserve some attention, as the control of exotic plants can depend on herbivore origin (Parker et al., 2006). Plant selectivity by herbivores is a multi-factorial choice and might combine several plant traits (morphotype, stoichiometric ratio, DMC, anatomical or structural traits, chemical defense compounds, toxins, deterrents, digestibility reducers, etc.), other than the one that we used (related to LDMC). Thus, the relative importance of each trait to palatability might differ depending on external conditions and/or the herbivore species. Unfortunately, only one or few herbivore species are compared in most of the studies on herbivory, while several herbivore species should be tested to measure response of the plant to herbivory in a general framework. Finally, an important step will be to test such a framework at a larger scale, allowing the herbivore to choose between different patches. Indeed, the positive effect of palatable species on the growth of less palatable species (associational susceptibility) can be blurred by higher patch attractiveness in the presence of palatable species.

## Conclusion

The main result of our study is that plant neighboring species and herbivores modestly interact in affecting plant traits involved in their dispersal strategies and establishment success. Furthermore, we found that (i) the response to crayfish presence and to the identity of neighboring species seemed to be species specific, and (ii) crayfish enhance the fragmentation rate (putatively related to plant regeneration/propagation) of the two exotic macrophytes *L. grandiflora* and *E. densa* in the presence of the native *M. spicatum*. Thus, the exotic crayfish could indirectly facilitate the invasion success of these exotic macrophytes.

To conclude, our paper presents some of the first results on associational resistance/susceptibility and lays the foundation for developing a general framework that combines plant community ecology and biological invasion ecology which could explain invasive species success. We showed that an important future step in the field of biological invasion is a better understanding of the response of plant traits to a set of different environmental constraints considered simultaneously. The new exotic–exotic interactions and associational resistance/susceptibility to herbivory should be taken into account to better understand exotic species establishment in the native recipient communities and their consequences.

## Ethics Statement

The experiment followed the French law (Directive 2010/63/UE) for animal manipulation.

## Author Contributions

GT and JH initiated the research project. LT and GT defined the research question and designed the experiments. LT conducted the experiments. BG analyzed the data. LT and BG interpreted the results and wrote the paper with contributions from all the authors. All authors have approved the manuscript.

## Conflict of Interest Statement

The authors declare that the research was conducted in the absence of any commercial or financial relationships that could be construed as a potential conflict of interest.
