# Supplementary material for: Response of Macrophyte Traits to Herbivory and Neighboring Species: Integration of the Functional Trait Framework in the Context of Ecological Invasions
Source: Front Plant Sci. 2019 Jan 10;9:1981. doi: 10.3389/fpls.2018.01981 (PMC6335397; doi:10.3389/fpls.2018.01981)
Supplement: Supplementary file 1 [file Data_Sheet_1.PDF]

Supplementary Information: Outlier detection

Response of macrophyte traits to herbivory and neighbouring species: integration of the functional trait framework in the context of ecological invasions

Lise Thouvenot[1,2], Benoit Gauzens[1,3], Jacques Haury[4,5], and Gabrielle Thiébaut[6]

[1]German Centre for Integrative Biodiversity Research (iDiv) Halle-Jena-Leipzig, Deutscher Platz 5e, 04103 Leipzig, Germany
[2]Institute of Biology, Leipzig University, Leipzig, Germany
[3]Institute of Biodiversity, University of Jena, Dornburger Str. 159, 07743 Jena, Germany
[4]INRA, UMR ESE, 35042 Rennes Cedex, France
[5]Agrocampus Ouest, 35042 Rennes Cedex, France
[6]Univ Rennes, CNRS, ECOBIO UMR 6553, F-35000, Rennes, France

Appendix S1. Outlier detection method

Several points appeared to have a significant impact on the conclusions of the statistical analyses. These potential outliers were first assessed from the visual inspection of data distribution among treatments and of 'residual plots' representing the relationship between the distribution of the standardized residuals of the model and predicted values. When this inspection suggested some potential influential points, we confirmed this visual impression by running an cross validation based on the Cook distance value of each point from

the dataset. The cook distance is a measure quantifying the influence of a data point on the outputs of linear models [1]. It is based on the differences between predictions made by the model for the dataset with and without the considered point.
Data points were considered as outliers in a dataset when their cook value were higher than six times the mean of the distribution of cook distance from this dataset. This criteria a bit more conservative than the usual multiplication of three [2]. Each outlier identified were then carefully inspected in the raw data.
Outliers were detected in:

- *L.grandiflora:* leaf dry matter content (LDMC) and surface leaf area (SLA).
- *E.densa:* leaf dry matter content.
- *M. spicatum:* relative growth rate (RGR)

In the following we will, for each of these three species, present all the diagnostic plots and argumentation explaining our choices to remove or not data points. All analyses have been made using R 3.4.3. We used the library R2jags 0.5-7 to run the MCMC of bayesian models with 70,000 iterations and a burn-in period of 50,000. The code of our analyses can be found at <git link>.

Appendix S2. Analyses for *L. grandiflora*

The plots of residual distributions for the different traits analysed led us to consider three suspicious data points in the values measured for LDMC and SLA. In the case of SLA, two of them was considered as outlier based on their cook distance, the third one being between the conventional and conservative threshold (Fig. 1). However, these three points all came from the same experimental unit and had incoherent values (SLA of 97.85, 234, and 259 $mm^2.mg^{-1}$), strongly arguing in favour of an issue with this replicate. We thus decided to remove this experimental unit from our analyses.

In the case of the LDMC, the cook distance distribution revealed three outliers with values of 15.63, 19.41 and 54.18 $mg.g^{-1}$ (Fig. 2). Two of them came from the experimental unit removed for the analysis of SLA. As before, the last data point from this unit has a cook distance value being between the conservative and the conventional thresholds. As what has been done for the LDMC, we estimated more secure to remove this experimental unit from the analysis.

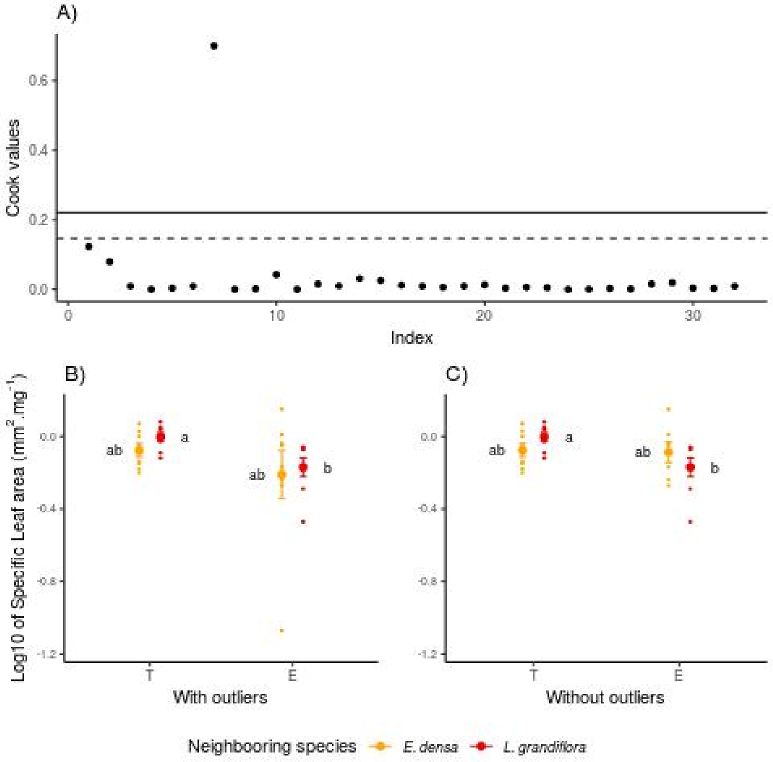

Figure 1: Oulier detection for the SLA of L. grandiflora: A) distribution of data points' Cook distances for the model fitted. Dashed line represents the usual detection threshold and solid line the more conservative threshold. Plots B) and C) are representation of the dataset when the outliers are included (B) and removed (C). Large points represent mean values and error bars set the standard deviation

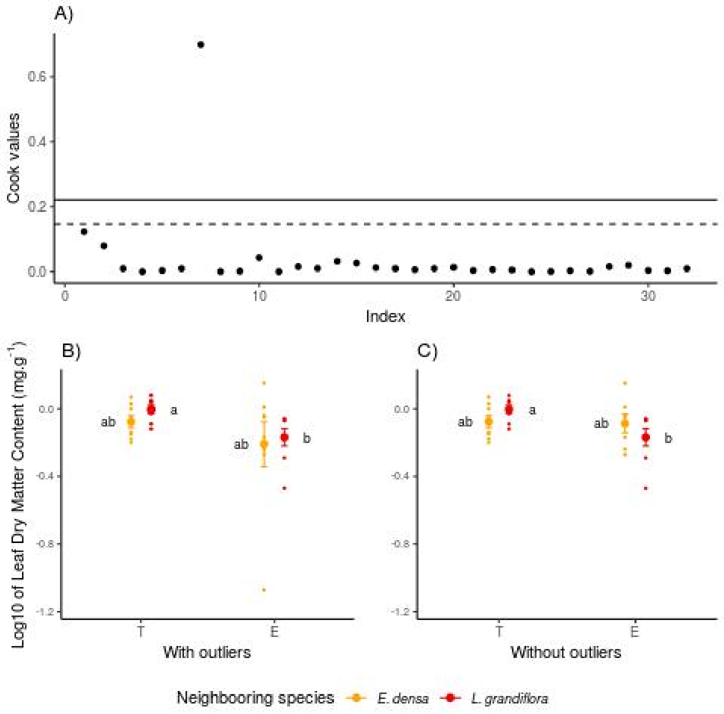

Figure 2: Oulier detection for the LDMC of L. grandiflora: A) distribution of data points' Cook distances for the model fitted. Dashed line represents the usual detection threshold and solid line the more conservative threshold. Plots B) and C) are representation of the dataset when the outliers are included (B) and removed (C). Large points represent mean values and error bars set the standard deviation

Appendix S3. Analyses for *E. densa*

Inspection of residual distributions for the different traits analysed led us to consider one point from the LDMC measurement as suspicious (value of 526.32 $mg.g^{-1}$). This impression was thereafter confirmed by the distribution of Cook's distances (Fig. 3).

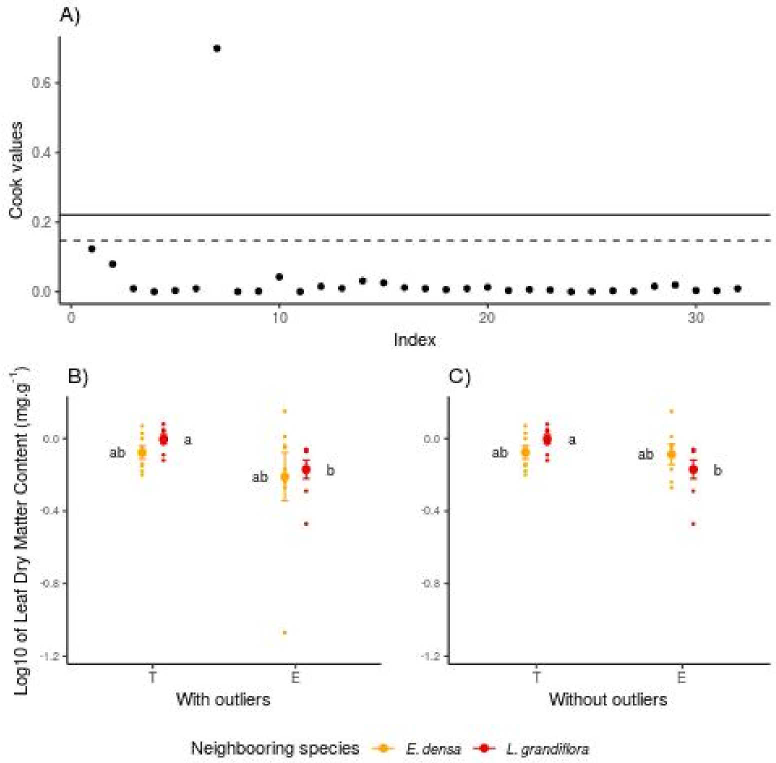

Figure 3: Oulier detection for the LDMC of E. densa: A) distribution of data points' Cook distances for the model fitted. Dashed line represents the usual detection threshold and solid line the more conservative threshold. Plots B) and C) are representation of the dataset when the outliers are included (B) and removed (C). Large points represent mean values and error set bars the standard deviation

Appendix S4. Analyses for *M. spicatum*

The plots of residual distributions for the different traits analysed led us to consider one suspicious data point in the values measured for the RGR of *M. spicatum* (-1.07 d^{-1}, Fig. 4).

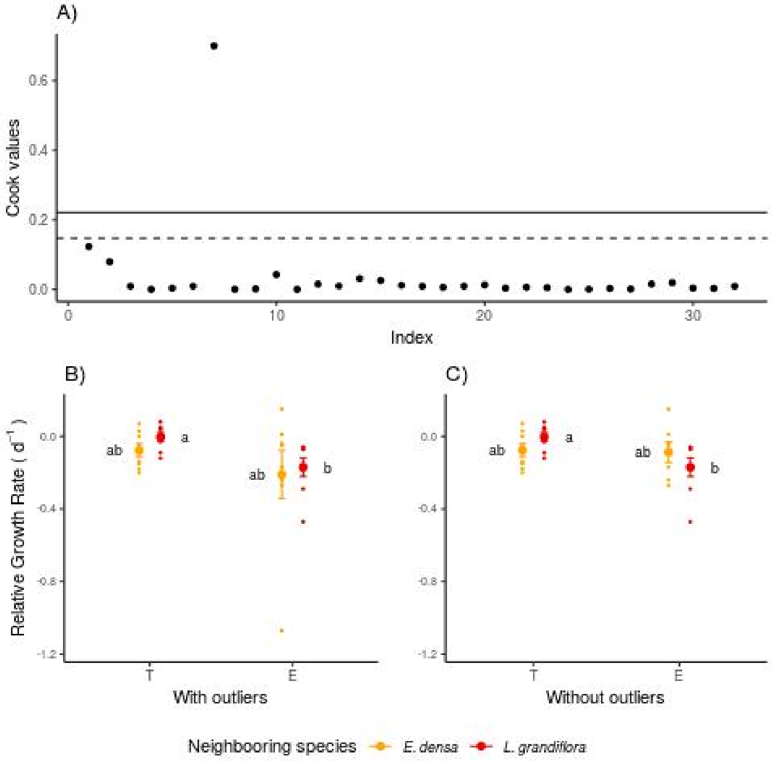

Figure 4: Oulier detection for the RGR of M. spicatum: A) distribution of data points' Cook distances for the model fitted. Dashed line represents the usual detection threshold and solid line the more conservative threshold. Plots B) and C) are representation of the dataset when the outliers are included (B) and removed (C). Large points represent mean values and error bars set the standard deviation

References

[1] R. Dennis Cook. Detection of influential observation in linear regression. *Technometrics*, 19(1):15–18, 1977.

[2] Siddu P. Algur Jyoti G. Biradar. Cooks distance and mahanabolis distance outlier detection methods to identify review spam. *International Journal Of Engineering And Computer Science*, 6(6), Jun. 2017.
